# Assessing the Effects of Cooking Fuels on *Anopheles* Mosquito Behavior: An Experimental Study in Rural Rwanda

**DOI:** 10.4269/ajtmh.21-0997

**Published:** 2022-02-21

**Authors:** Ian Hennessee, Miles A. Kirby, Xavier Misago, Jackie Mupfasoni, Thomas Clasen, Uriel Kitron, Joshua P. Rosenthal, Emmanuel Hakizimana

**Affiliations:** ^1^ Gangarosa Department of Environmental Health, Rollins School of Public Health, Emory University, Atlanta, Georgia;; ^2^ Department of Global Health and Population, Harvard T.H. Chan School of Public Health, Harvard University, Boston, Massachusetts;; ^3^ Malaria and other Parasitic Diseases Division, Rwanda Biomedical Center, Ministry of Health, Kigali, Rwanda;; ^4^ Department of Environmental Sciences, Emory University, Atlanta, Georgia;; ^5^ Fogarty International Center, National Institutes of Health, Bethesda, Maryland

## Abstract

Globally, cleaner cooking fuels are increasingly promoted to reduce household air pollution. However, there is concern that reductions in smoke from biomass fuels could lead to more favorable conditions for mosquitoes and potentially increase vectorborne disease risk. We investigated household entry, host-seeking, household exit, and mortality among *Anopheles* mosquitoes across three cooking fuel types: wood, charcoal, and liquid petroleum gas (LPG) in six experimental huts in Rwanda. Fifty laboratory-reared *Anopheles gambiae* mosquitoes were released each night in entry compartments outside each hut, and fuels were burned for 1 hour in the hut verandas. Collectors conducted human landing catch during cooking and for 2 hours afterward, and CDC light traps were used for the rest of the night to measure host-seeking. Differences in each outcome were assessed using generalized linear mixed models with random effects for hut, collector, and day. Cooking with LPG compared with wood and charcoal was associated with substantial increases in household entry and host-seeking. Household exit was not significantly different across fuels, and mortality was lower in LPG-burning huts compared with wood. Although these results are not directly generalizable to field conditions, they indicate a potential for clean fuel adoption to increase exposure to *Anopheles* mosquitoes compared with traditional biomass fuels. Additional entomological and epidemiological studies are needed to investigate changes in disease vector exposure associated with clean fuel adoption, and evaluate whether enhanced vector control interventions should be promoted in tandem with cleaner cooking fuels.

## INTRODUCTION

Cleaner cooking fuels such as liquid petroleum gas are increasingly promoted to reduce household air pollution (HAP), which is responsible for more than 2.3 million deaths per year. ^1^^,^ ^2^ However, there is some concern that reductions in smoke or other volatiles from traditional fuels could affect mosquito behavior and transmission of malaria or other vectorborne diseases. ^3^ Smoke has been used as an insect repellent for centuries, ^4^ and components of biomass combustion such as carbon dioxide (CO_2_), heat, and chemical volatiles are known to influence mosquito behavior. ^5^^–^ ^7^

Numerous entomological studies have reported negative associations of biomass combustion with density and household entry of *Anopheles* mosquitoes. ^8^^–^ ^11^ Biomass combustion is also associated with reduced blood feeding success, altered resting behavior, and higher exit rates of mosquitoes in experimental and observational field settings. ^8^^,^ ^12^^–^ ^17^ However, these studies were not designed to measure the effects of biomass fuel combustion for cooking or domestic heating, or compare the effects of different cooking fuels.

Although epidemiological evidence is limited, a cluster randomized controlled trial of cleaner-burning biomass stoves in Malawi reported a significant increase in malaria incidence among children in houses that received the intervention. ^18^ A recent case–control study in Guatemala also found that individuals from houses that cooked with fuels other than firewood had an increased risk of arbovirus infection compared with houses that cooked with firewood in the main house or on open hearths. ^19^ In both cases, however, these were secondary outcomes of health impact evaluations. Other observational studies have reported mixed associations between biomass fuel use and malaria incidence. ^20^^–^ ^27^

Despite this entomological and epidemiological evidence, no studies have directly investigated the impacts of the adoption of clean-burning cooking fuels on mosquito behavior or vectorborne disease transmission. ^3^ This information is critical for understanding potential effects of clean fuel adoption and, if necessary, recommending the promotion of vector control measures in tandem with clean cooking interventions. As a preliminary step in addressing this research gap, we undertook an experimental evaluation of the impacts of traditional and clean cooking fuels on the behavior of the most important malaria vector in Rwanda, *Anopheles gambiae.*

## MATERIALS AND METHODS

### Research objectives.

The primary objective of this study was to evaluate if, and to what extent, the adoption of clean-burning fuels could affect *Anopheles* mosquito behavior. We used a series of controlled, semi-field experiments to measure differences in household entry, host-seeking, household exiting, and mortality among *Anopheles* mosquitoes across three commonly used fuel types: wood, charcoal, and liquid petroleum gas (LPG).

### Study location.

This study was conducted in Eastern Province, Rwanda. The area was selected in part because of its proximity to a large randomized controlled trial to assess the health effects of cooking with LPG in a population traditionally relying on solid biomass fuels. ^28^ Eastern Province has the highest malaria burden of any part of the country, ^29^ and malaria prevalence among children aged under 5 years increased from 3.4% to 18.4% between 2010 and 2017. ^30^ The *An. gambiae* species complex are the principal malaria vectors in Eastern Province and elsewhere in Rwanda. ^31^

### Experimental huts.

The Rwanda Biomedical Center (RBC) maintains a group of six experimental huts near the town of Ruhuha in Bugesera District, Eastern Province. Experimental huts are typically used to test vector-control methods and are identically constructed and situated close together to reduce potential confounding from environmental conditions such as ambient temperature or humidity. ^32^ The Rwanda experimental huts are constructed in the West Africa style with concrete block walls, corrugated metal roofs, and a water-filled moat around the perimeter to deter ants. They have four small windows around the outside and a screened veranda in the upper section of the back wall. They do not have chimneys, but emissions from indoor cooking can escape from windows, a screened veranda, and gaps between the walls and roof. Three of the RBC huts had cement walls and three had mud walls. The main room dimensions were 1.75 × 2.5 m, with a ceiling height of approximately 2 m. The adjacent verandas were approximately 1.5 × 1.5 m.

### Trap design and hut modifications.

This study necessitated slight modifications to the experimental huts, which are typically designed to allow entry of wild mosquitoes but do not have compartments for introducing laboratory-raised mosquitoes or trapping mosquitoes that exit the huts. We worked with a local team of tailors and welders to build and install entry compartments and window exit traps. Designs for both entry compartments and exit traps were adapted from window entry and exit traps, as described by Okumu et al. (2012), Diabaté et al. (2013), and the WHO (2013) for use in *Anopheles* mosquito sampling and trapping in experimental huts. ^32^^–^ ^34^

**Figure 1.**
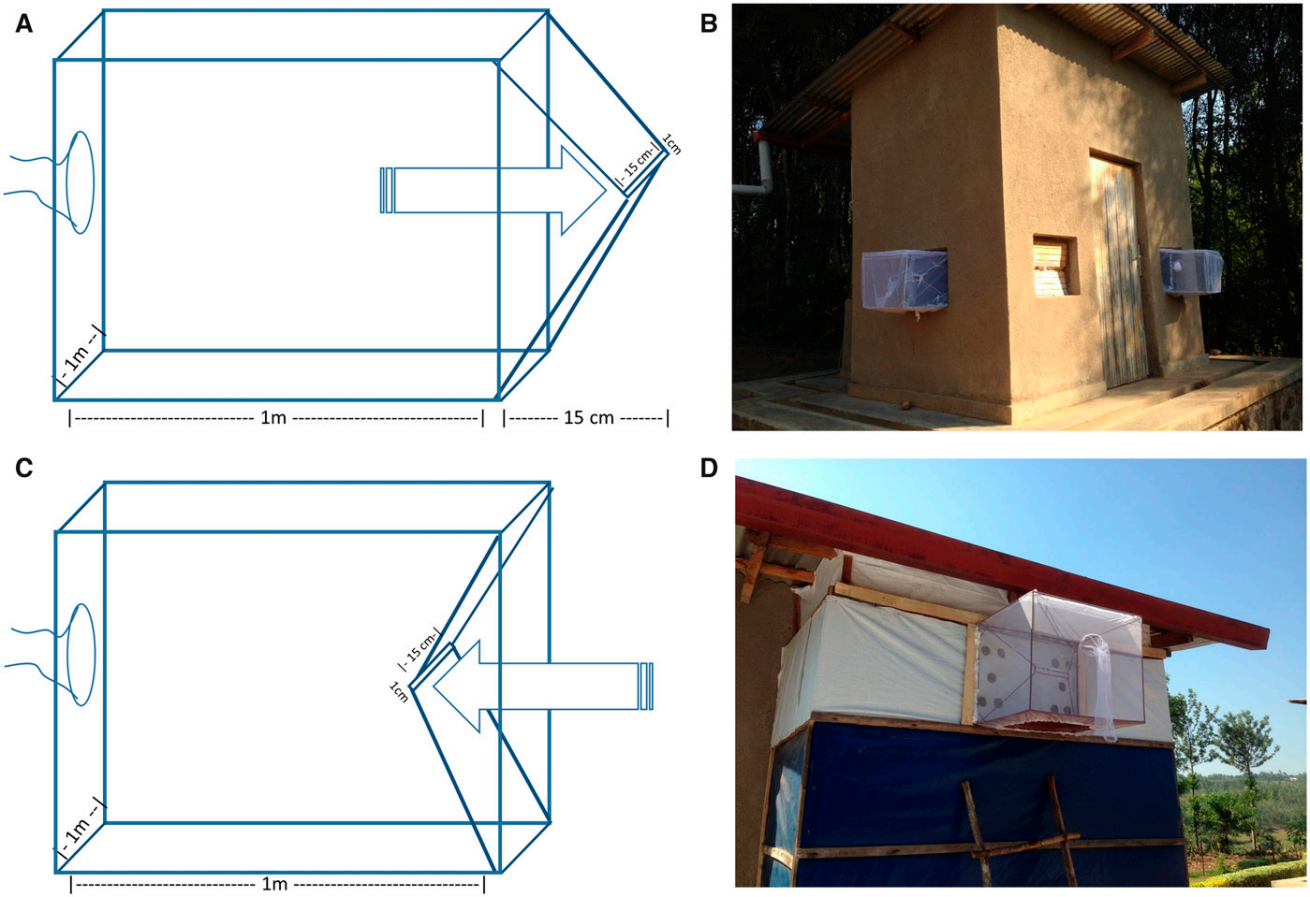
Design and layout of entry compartments and exit traps. Design of entry compartment (**A**) and window exit traps (**C**). Entry compartments were mounted to the right of the front door of each hut, which faced east (**B**). Exit traps were mounted to the south wall of each hut (**B**), as well as the upper veranda area in the back of each hut (**D**). This figure appears in color at www.ajtmh.org.

Entry compartments were constructed of 1 × 1 × 1 m rigid steel frames wrapped in untreated mosquito netting. A convex trapezoidal prism extended 15 cm into the interior of the hut, with a 1 cm high × 50 cm wide opening slit (Figure 1A). Collectors used manual aspirators to introduce mosquitoes through a 10-cm diameter baffle on the outside of each compartment, which otherwise was tied off to prevent mosquitoes from escaping. Mosquitoes could then pass through the opening slit into the hut, but once inside the hut, the convex design prohibited them from returning back into the entry compartment. This entry compartment design has been shown to be effective in ensuring that mosquitoes can enter a space through the opening at the end of the convex prism but cannot return back the same way. ^33^^,^ ^35^ Window exit traps were almost identical to the entry compartments, but were fitted with a concave trapezoidal prism instead of a convex prism (Figure 1C). The concave side of the window traps were fixed to the windows of the hut such that mosquitoes could fly out of the hut and into the window exit trap but could not return the other direction.

One entry compartment was installed on each hut via an open window to the right of the front door (Figure 1B). Any remaining gaps between the frame of the compartment and the window frame were sealed with cotton to prevent escape. Two window exit traps were installed on each hut. One was installed on a lower window on the left, south-facing wall of each hut (Figure 1B). Another window exit trap was installed on the uppermost section of the veranda, in the back wall of each experimental hut (Figure 1D). Typically the veranda section of the experimental huts is screened to mimic a semioutdoor setting. ^36^ However, because our experiment was designed to investigate the effects of indoor cooking on mosquito behavior, we sealed the screening with plastic tarp so the whole hut was more representative of indoor conditions. We then installed 10 holes (5-cm diameter) in the tarp to mimic ventilation blocks, or claustras, which are commonly used in Rwandan concrete block and mud-brick houses for ventilation. The veranda exit traps were placed over the ventilation holes (Figure 1D).

### Laboratory methods for raising mosquitoes.

*An. gambiae* s.s., Kisumu strain mosquitoes were raised in Kigali at the entomology laboratory of the RBC—Malaria and Other Parasitic Diseases Division (MOPDD). Larvae were reared in distilled water and fed with a 10% liver powder solution, and emerging adults were kept in holding cages at 26–28°C and 70% to 80% relative humidity (RH). Non–blood-fed, 3- to 5-day-old females were collected using manual aspirators and transported to the study site in mesh-covered cups with a 10% sugar solution on cotton wool pads. Each cup was held in a cooler before use.

### Fuels tested.

We tested three cooking fuels: wood, charcoal, and LPG. Wood is the most commonly used domestic cooking fuel in Rwanda and is used by 63% of households as their primary cooking fuel. Charcoal is the second most commonly used cooking fuel Rwanda and is used by more than 17% of households. ^29^ We used locally sourced, dried eucalyptus firewood, which is the most widely cultivated fuel-wood species. ^37^ We sourced local charcoal made from eucalyptus wood. Finally, although LPG is not yet widely used in Rwanda, it is increasingly promoted to reduce the harmful health effects of household air pollution primarily due to cooking with solid biomass fuels. ^38^^–^ ^40^ Its use for cooking has increased from 0.1% of Rwandan houses in 2010 to 1.6% in 2017, and it is slated to become a major source of cooking fuel in the next decade. ^29^^,^ ^41^^,^ ^42^ Globally, nearly three billion people still rely on traditional biomass fuels such as wood and charcoal for cooking and heating. However, LPG use is expanding rapidly in many low- and middle-income countries. ^43^^,^ ^44^

### Schedule and timeline of experiments.

We conducted three phases of experiments. In phase 1, we conducted 6 days of baseline testing in which collections were performed in the absence of any cooking fuel. This phase served as a baseline metric for household entry, host-seeking, and exiting, and to ensure that there were no systematic differences between the huts.

In phase 2 we used a modified Latin Square design in which collectors rotated between each experimental hut each night, but fuels were held constant for each hut. This was to address the potential residual effects from the combustion of certain fuels, especially wood fires. ^8^ Collectors rotated huts each night to address the potential for differing biting attractiveness or different practices between collectors. Phase 2 consisted of three full rotations of the collectors across the six huts, for 18 total days of collection.

In phase 3, we conducted a true Latin Square design, in which LPG and wood fuels were rotated each night to reduce the potential effects of ambient environmental differences. ^34^ Collectors remained in the same hut each night. Phase 3 lasted 6 days and used two iterations of a 2 × 3 Latin Square layout to obtain a fully balanced sample. Otherwise, all methods as described subsequently were identical for each phase.

### Experimental procedures.

**Figure 2.**
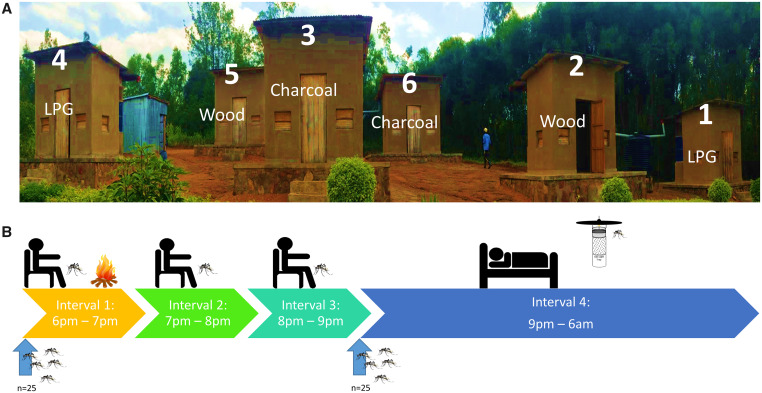
Experimental hut layout and experimental design. (**A**) The layout of the experimental huts in Bugesera District, Rwanda, with example cooking fuel designations. (**B**) Experimental procedures: collectors cooked and conducted human landing catch for 1 hour between 6 pm and 7 pm (Interval 1). They then extinguished cooking fires and conduct human landing catch for 2 more hours until 9 pm (Intervals 2 and 3). They then retired under an untreated bed net, and CDC Light traps were used for the rest of the night until 6 am the following morning (Interval 4). Twenty-five *An. gambiae* mosquitoes were released at 6 pm, and another 25 were released at 9 pm. Household entry, host-seeking, and household exit were recorded at the end of each interval. Mortality was recorded only at the end of each complete sampling round. This figure appears in color at www.ajtmh.org.

Before the study, the huts were randomly assigned to LPG, wood, or charcoal fuels so that there were two huts for each fuel type (Figure 2A). These fuel assignments were held constant throughout phase 2. For phase 3, only LPG and wood fuels were used. They were randomly assigned to each hut for the first night and rotated nightly thereafter. This decision was made to maximize our ability to compare the effects of LPG with wood, which is the most commonly used cooking fuel in Rwanda.

Wood fires were lit in traditional three-stone stoves. Charcoal fires were lit in locally made, unimproved stoves call *imbaburas*. ^45^ Ten-kg LPG fuel cylinders were purchased locally and fitted with a simple burner and an attachment for cooking on top of the cylinder.

Each sampling round lasted from approximately 6 pm to 6 am the next morning. No fuels were lit during phase 1. During phases 2 and 3, each fuel was lit in the veranda area of the experimental huts at approximately 5:45 pm each night, and a 2-L pot of water placed above the flame and brought to a low rolling boil to standardize combustion intensity across fuels. Four kilograms of dried eucalyptus wood and 200 g of charcoal were premeasured and the wood chopped into 2- to 5-cm diameter pieces so it could be routinely replenished during cooking. The LPG stoves were lit and maintained at medium intensity. Each fuel source remained burning for 1 hour and was extinguished at 7 pm.

At 6 pm, trained entomology officers employed by the Rwanda Biomedical Center used manual aspirators to release 25 *An. gambiae* s.s. mosquitoes in entry compartments attached to the windows of each hut. Trained collectors sat on a small stool approximately 1 m from the stove and wore long-sleeved clothes with one pant-leg rolled up. They then conducted human landing catch using a flashlight and glass collection tubes to catch all mosquitoes that landed on them. ^31^ Human landing catch was used to measure host-seeking behavior during cooking (6 pm–7 pm) and for 2 hours afterward (7 pm–8 pm and 8 pm–9 pm) (Figure 2B, Intervals 1–3). Mosquitoes caught during each of the 3 hourly intervals were placed in prelabeled envelopes for counting the next day. At the end of each hour, entomology officers used flashlights to count and record the number of mosquitoes remaining in the entry traps, as well as in the two window exit traps outside each hut. Because all mosquitoes were laboratory-reared, there was no potential for pathogen transmission if collectors were incidentally bitten while conducing human landing catch.

At 9 pm the collectors were asked to retire under untreated bed nets. Twenty-five more mosquitoes were then released into the entry compartments of each hut to simulate the arrival of host-seeking mosquitoes long after cooking was completed. Host-seeking was approximated during the fourth interval (9 pm–6 am) via Miniature CDC light traps (Model 512, John W. Hock Company, Gainesville, FL). CDC light traps are frequently used as a proxy for host-seeking in settings where human landing catch is not ethical or feasible. ^46^^–^ ^48^ Light traps were hung at a height of approximately 1.5 m at the foot end of the bed, a height that has been shown to maximize catches of host-seeking *An. gambiae* s.l. mosquitoes. ^49^ The light traps were illuminated from 9 pm to 6 am the next morning to measure host-seeking during the night (Figure 2B, Interval 4). At 6 am the next morning the entomology officers made final counts of the number of mosquitoes remaining in the entry compartments and window traps outside each hut. They also recorded the number of mosquitoes found in CDC light traps, as well as all dead mosquitoes found in huts or entry traps.

Real-time fine particulate matter (PM_2.5_) concentrations, temperature (°C), and percent RH were measured inside of each experimental hut with Particle And Temperature Sensors (PATS+) devices (Berkeley Air Monitoring Group, Berkeley, CA). ^50^^,^ ^51^ Devices were clean air zeroed according to manufacturing instructions and set to provide PM_2.5_ readings every minute for the 12-hour duration of each study round. Devices have a PM_2.5_ lower detection limit of 10 to 50 μg/m^3^, and values below the lower end of this limit were recorded as 10 μg/m^3^. Likewise, values above the upper limit of detection of 25,000 to 50,000 μg/m^3^ were recorded as 25,000 μg/m^3^.

PATS+ devices were suspended on one wall in each hut next to the CDC light traps at 1.5 m, approximately 2 m from the cooking fuels. Devices were hung approximately 1.5 m from the closest window where the entry compartments were installed. Twelve-hour means, medians, and interquartile ranges of PM_2.5_, temperature, and RH were calculated to represent average levels during each sampling round, and interval-specific means were also calculated.

### Primary outcomes.

Primary outcomes included percent household entry, host-seeking, household exit, and mortality. These were measured cumulatively during each sampling round, as well as during individual sampling intervals (Interval 1 = 6 pm–7 pm, Interval 2 = 7 pm–8 pm, Interval 3 = 8 pm–9 pm, and Interval 4 = 9 pm–6 am).

#### Household entry.

Cumulative household entry ( $HE_{\mathrm{cumu}}$) was defined as the proportion of mosquitoes that entered each hut during each round of sampling over the total number of mosquitoes released (*N* = 50). Interval-specific household entry $HE_{i}$ was calculated as the proportion of mosquitoes that entered each hut by the end of each interval, *i* (*i =* 1, 2, … 4), out of the number remaining in entry compartments at the start of that interval.

$$HE_{\mathrm{cumu}}=\frac{\mathrm{total}\;\mathrm{entered}\;}{50}$$

$$HE_{i}=\frac{\mathrm{entered}\;\mathrm{during}\;\mathrm{interva}l_{i}}{\mathrm{remaining}\;at\;\mathrm{start}\;\mathrm{of interva}l_{i}}$$

#### Host seeking.

Cumulative host seeking ( $HS_{\mathrm{cumu}}$) was measured as the proportion of mosquitoes that sought a host during each sampling round over the total number of mosquitoes released (*N* = 50). Interval-specific host-seeking ( $HS_{i}$) was calculated as the proportion of mosquitoes that sought a host during each interval over the number that had not sought a host before the start of that interval, including mosquitoes remaining in entry compartments at the start of the interval, as well as those that had entered huts but not sought a host during prior interval(s). During the first three intervals, host-seeking was measured directly via human landing catch; light traps were used to approximate host-seeking during the fourth interval to reduce collector fatigue.

$$HS_{\mathrm{cumu}}=\frac{\mathrm{total}\;\mathrm{sought}\;\mathrm{host}}{50}$$

$$HS_{i}=\frac{\mathrm{sought}\;\mathrm{host}\;\mathrm{during}\;\mathrm{interva}l_{i}}{\begin{array}{c} \#\mathrm{remaining}\;in\;\mathrm{entry}\;\mathrm{compartment}\;at\;\mathrm{start}\;of\\ \;\mathrm{interva}l_{i}+(\#\;\mathrm{entered}\;\mathrm{during}\;\mathrm{prior}\;\mathrm{intervals}\\ -\#\;\mathrm{sought}\;\mathrm{host}\;\mathrm{during}\;\mathrm{prior}\;\mathrm{intervals})\; \end{array}}$$

#### Household exit.

Cumulative household exit ( $HE{\text{X}}_{\mathrm{cumu}}$) was defined as the proportion of mosquitoes that exited the hut via the two window exit traps during each sampling round over the total number released (*N* = 50). Interval-specific household exit ( $HEX_{i}$) was defined as the ratio of mosquitoes that exited huts into exit traps during each sampling interval divided by the sum of the number that entered huts over the course of the interval plus those that had entered and not exited during the prior interval(s).

$$HEX_{\mathrm{cumu}}=\frac{\mathrm{total}\;\mathrm{exited}\;}{50}$$

$$HEX_{i}=\frac{\mathrm{exited}\;\mathrm{during}\;\mathrm{interva}l_{i}}{\begin{array}{c} \#\;\mathrm{enterted}\;\mathrm{during}\;{\mathrm{interval}}_{i}+\\ (\#\;\mathrm{entered}\;\mathrm{during}\;\mathrm{prior}\;\mathrm{intervals}\\ -\#\;\mathrm{exited}\;\mathrm{during}\;\mathrm{prior}\;\mathrm{intervals}) \end{array}}$$

#### Mortality.

Cumulative mortality ( $\mathrm{Mor}t_{\mathrm{cumu}}$) was calculated by counting the number of dead mosquitoes inside the hut as well as in entry compartments and window traps. Mortality was only measured at the end of each sampling round, instead of at each interval due to challenges with locating dead mosquitoes in the dark. Percent mortality was calculated as the number of dead mosquitoes divided by the total number of mosquitoes released in each hut (*N* = 50).

$$(\mathrm{Mor}t_{\mathrm{cumu}})\;=\frac{\mathrm{total}\;\mathrm{dead}\;}{50}$$

### Statistical analysis.

We conducted all statistical analyses using R version 4.0.2 (R Core Team, Vienna, Austria) ^52^ and SAS version 9.4 (SAS Institute, Cary, NC). We fit generalized linear mixed effect models (GLMMs) with a logit link and a binomial distribution to estimate the impacts of each fuel type on the odds of household entry, host-seeking, household exit, and mortality. Because wood is the most commonly used cooking fuel in Rwanda, ^29^ we treated wood as the reference variable and included charcoal and LPG as separate dummy variables. To account for nonindependence of observations, we included random effects for hut, collector, and day. ^32^ We also included a fixed effect for wall-type using a dummy value for cement versus mud, with mud as the reference category. Primary analyses were first conducted for cumulative outcomes. Interactions between sampling interval and each outcome were assessed, and, if significant, effect measures were reported separately for each sampling interval. As a secondary analysis, we included the baseline results in the model and conducted pairwise comparisons across all groups using Tukey’s tests to account for multiple comparisons.

We then assessed potential dose-response effects of PM_2.5_, indoor temperature and RH on household entry, host-seeking, household exit, and mortality. We first fit linear mixed effect models with random effects for hut, collector, and day to model the change in each outcome per standard deviation increase in each predictor variable. We assessed the relative importance of PM_2.5_, temperature and RH via the change in adjusted *R*^2^ and Akaike information criteria (AIC) values when each variable was added last to the full model. We also analyzed potential nonlinear associations between the predictors and each outcome using generalized additive mixed effect models (GAMMs). Analyses were conducted separately for phases 1, 2 and 3. However, the results for phases 2 and 3 were nearly identical and were therefore pooled for the final analysis.

### Ethics.

The study was reviewed and approved by the Rwanda National Ethics Committee under Institutional Review Board (IRB) 00001497, No. 194/RNEC/2019. The Emory IRB reviewed this study and determined it was exempt from IRB clearance because it did not involve research on human subjects.

## RESULTS

### Baseline.

During phase 1, 6 days of collections were conducted in the absence of any fuel use to estimate baseline parameters for each outcome and to ensure comparability across each of the six huts. Cumulatively, a mean of 67.9% released mosquitoes entered huts and 41.3% sought a host (Table 1). Of the mosquitoes that entered huts, a mean of 11.9% exited. Household entry and host-seeking were generally highest during the first and fourth sampling intervals, and household exit peaked during the fourth sampling interval. Mortality was low, averaging 4.2% of all mosquitoes released. One-way analysis of variance tests showed no significant differences in any of the four outcomes across the six experimental huts (*P* > 0.05). Wall type (cement versus mud) was not a significant predictor of any outcome.

**Table 1:** Percent household entry, host-seeking, household exit and mortality of released mosquitoes during baseline tests with no fuels (phase 1), mean (standard deviation)

	Sampling intervals			
Cumulative	6 pm–7 pm	7 pm–8 pm	8 pm–9 pm	9 pm–6 am
67.9 (23.5)	43.1 (26.7)	30.8 (23.4)	27.2 (29.2)	56.3 (27.2)
41.3 (19.4)	23.2 (23.9)	21.4 (21.6)	16.7 (16.5)	21.6 (14.3)
11.9 (12.7)	2.3 (4.9)	8.6 (24.7)	27.8 (40.0)	19.7 (21.7)
4.2 (4.4)	–	–	–	–

Mortality was only measured once at the end of each sampling round, so interval-specific results are not reported.

### Household entry.

**Figure 3.**
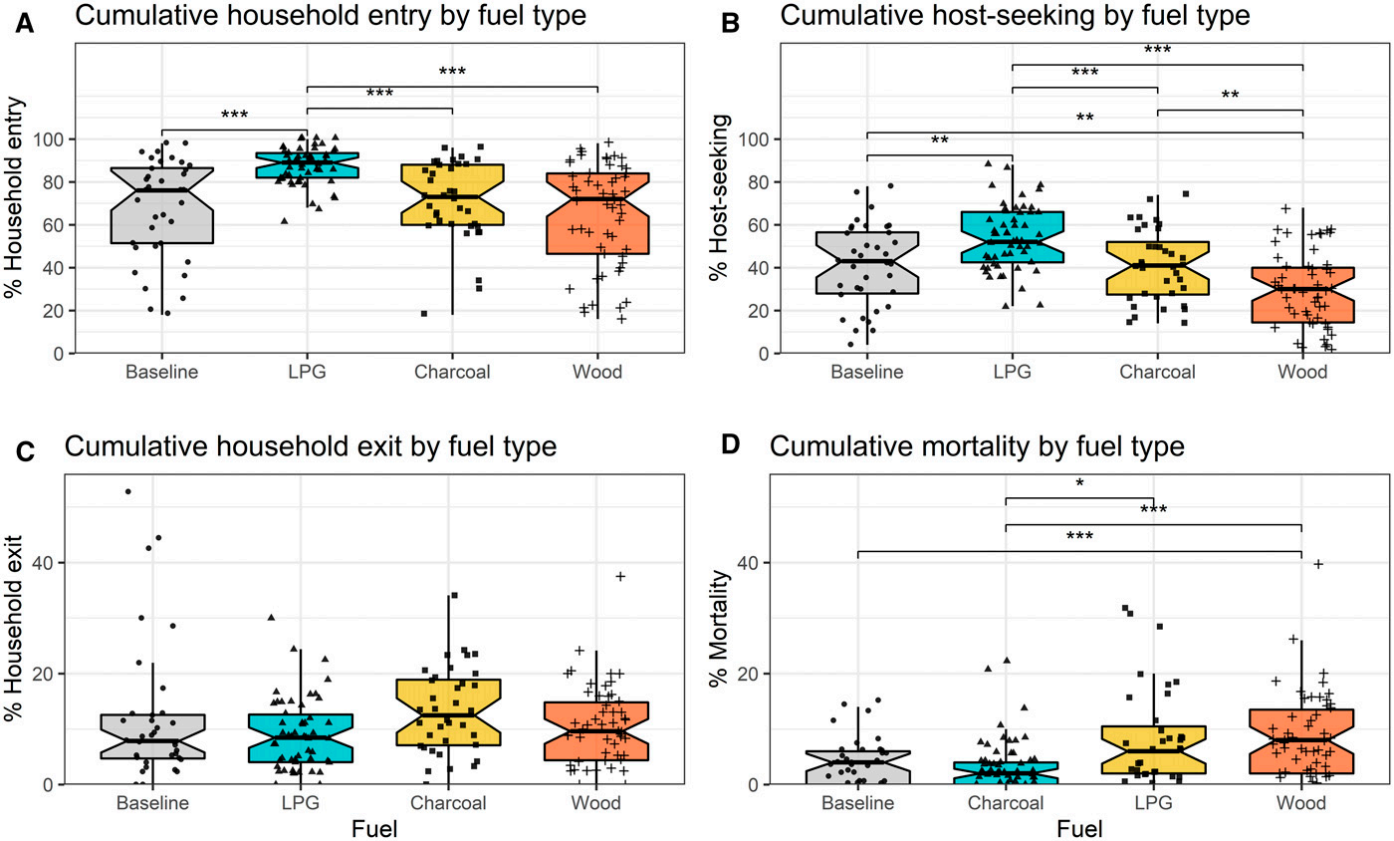
Cumulative household entry, host-seeking, household exit, and mortality by fuel type. Box plots show each outcome as a percent of all mosquitoes released. Points overlaid on boxplots are individual measurements for each sampling round. Asterisks depict significant differences between fuel types after adjusting for multiple comparisons: *P < 0.05, **P < 0.01, and ***P < 0.001. This figure appears in color at www.ajtmh.org.

When fuels were introduced, the cumulative proportion of mosquitoes that entered houses ranged from 65.1% in huts cooking with wood to 71.1% in charcoal and 87.3% in LPG huts. Overall, the odds of household entry were 2.7 (95% confidence interval [CI]: 2.1–3.3) times higher in LPG-burning huts compared with wood, and 1.7 (95% CI: 1.2–2.4) times higher in charcoal-burning huts compared with wood (Table 2). After applying Tukey’s test for multiple comparisons, household entry in LPG huts was also significantly higher than in charcoal huts and baseline huts where no fuel was used (Figure 3A).

**Table 2:** Cumulative household entry, host-seeking, household exit, and mortality as a percent of all mosquitoes released

	Fuel	Mean (SD)	OR (95% CI)	*P* value
Household entry	LPG	87.3 (8.6)	2.7 (2.1–3.3)	< 0.001***
	Charcoal	71.1 (18.6)	1.7 (1.2–2.4)	0.002**
	Wood (ref)	65.1 (23.9)	1 (NA–NA)	NA
Host seeking	LPG	54 (14.9)	2.5 (2.1–2.9)	< 0.001***
	Charcoal	41.1 (16.8)	1.7 (1.3–2.3)	< 0.001***
	Wood (ref)	29.7 (17.9)	1 (NA–NA)	NA
Household exit	LPG	8.9 (6.5)	0.8 (0.6–1.1)	0.246
	Charcoal	13.3 (7.7)	1.4 (0.9–2.1)	0.088
	Wood (ref)	10.3 (7.3)	1 (NA–NA)	NA
Mortality	LPG	3.8 (4.6)	0.6 (0.4–0.8)	0.001**
	Charcoal	8.1 (8.8)	0.9 (0.6–1.4)	0.607
	Wood (ref)	8.7 (7.5)	1 (NA–NA)	NA

LPG = liquid petroleum gas; NA = not applicable; OR = odds ratio; ref = reference variable; SD = standard deviation. For all regressions, wood was treated as the reference variable and LPG and charcoal were included as separate dummy variables.

**Figure 4.**
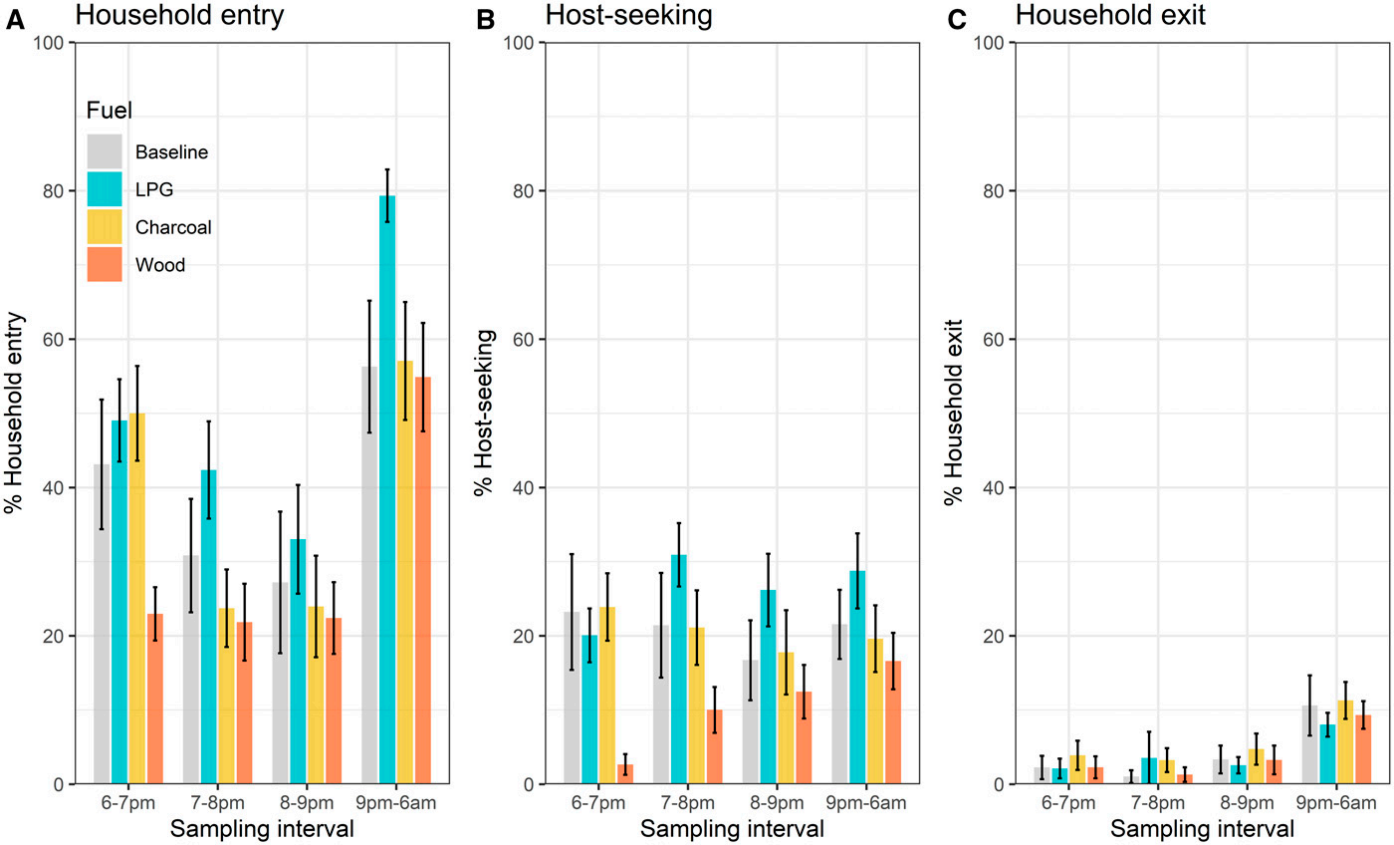
Interval-specific household entry, host-seeking, and household exit by fuel type. The first plot (**A**) shows mean household entry during each sampling interval as a percent of all mosquitoes remaining in entrance compartments at the start of each interval. The second plot (**B**) shows mean host-seeking during each sampling interval as a percent of all mosquitoes that had not sought a host by the start of each interval. The third plot (**C**) shows mean household-exit during each sampling interval as a percent of all mosquitoes that entered houses and did not seek a host by the end of each interval. Error bars represent 95% confidence intervals for each mean. This figure appears in color at www.ajtmh.org.

Sampling interval was a significant effect modifier of the relationship between household entry and fuel type (*P* < 0.01). The difference was particularly pronounced during cooking (6 pm–7 pm), when the odds of household entry were 3.3 (95% CI: 2.6–4.0) times higher in LPG and 3.5 (95% CI: 3.4–4.6) times higher in charcoal huts, compared with wood (Table 3). These differences were less pronounced in later intervals, although household entry remained higher in LPG huts compared with wood and charcoal huts during all subsequent intervals (Figure 4A).

**Table 3:** Household entry, host-seeking, and household exit during each sampling interval

		Interval 1: 6 pm–7 pm		Interval 2: 7 pm–8 pm	
		Mean (SD)	OR (95% CI)	Mean (SD)	OR (95% CI)
Household entry	LPG	49 (20.8)	3.2 (2.6–4.0)***	42.4 (24.6)	2.8 (2.1–3.6)***
	Charcoal	50 (19.6)	3.5 (2.4–4.6)***	23.7 (16)	1.4 (0.9–2.2)
	Wood (ref)	23 (13.5)	–	21.8 (19.4)	–
Host-seeking	LPG	20.1 (13.6)	8.7 (5.7–13.3)***	30.9 (16)	3.9 (2.9–5.3)
	Charcoal	23.9 (13.9)	13.0 (7.8–21.5)***	21.1 (15.4)	2.36 (1.48–3.75)
	Wood (ref)	2.7 (5.3)	–	10 (11.6)	–
Household exit	LPG	2.1 (5)	0.5 (0.2–1.1)	9.6 (22.7)	4.8 (1.4–17.4)*
	Charcoal	3.9 (6)	0.9 (0.3–2.3)	21.9 (35.7)	4.7 (0.8–19.7)*
	Wood (ref)	2.3 (5.5)	–	5.1 (16.8)	–

		Interval 3: 8 pm–9 pm		Interval 4: 9 pm–6 am	
Household entry	LPG	33 (27.4)	1.1 (0.7–1.7)	79.3 (13.3)	2.4 (1.9–3.1)***
	Charcoal	24 (20.9)	0.6 (0.3–1.0)	57.1 (24.3)	1.5 (1.0–2.2)*
	Wood (ref)	22.4 (18.2)	–	54.9 (27.4)	–
Host-seeking	LPG	26.2 (18.4)	2.1 (1.5–3.1)***	28.8 (19)	2.0 (1.6–2.5)***
	Charcoal	17.8 (17.4)	1.2 (0.7–2.0)	19.6 (13.8)	1.2 (0.8–1.7)
	Wood (ref)	12.5 (13.5)	–	16.6 (14.3)	–
Household exit	LPG	28.9 (42.1)	1.8 (0.9–4.2)	14.7 (11.8)	0.9 (0.6–1.2)
	Charcoal	41.9 (46.3)	5.3 (2.4–13.7)***	24 (19.5)	1.5 (0.9–2.4)
	Wood (ref)	16.2 (34.1)	–	16.8 (15.2)	–

LPG = liquid petroleum gas; OR = odds ratio; ref = reference; SD = standard deviation. For all regressions, wood was treated as the reference variable and LPG and charcoal were included as separate dummy variables. *P < 0.05; **P < 0.01; ***P < 0.001.

### Host-seeking.

Cumulative host-seeking as a percent of all mosquitoes released each sampling round averaged 29.7% in wood, 41.1% in charcoal, and 54.0% in LPG-burning huts. Cooking with LPG was associated with 2.5 (95% CI: 2.1–2.9) times higher odds of host-seeking compared with wood, and the odds of host-seeking were 1.7 (95% CI: 1.3–2.3) times higher in charcoal compared with wood-burning huts (Table 2). Compared with baseline conditions, host-seeking was significantly higher in LPG and lower in wood-burning huts (Figure 3B).

Again, we observed a significant interaction between fuel-type and sampling interval (*P* < 0.001). The odds of host-seeking during cooking (6 pm–7 pm) were 8.7 (95% CI: 5.7–13.3) times higher in LPG and 13.0 (95% CI: 7.8–21.5) in charcoal compared with wood-burning huts (Table 3, Figure 4B). Host-seeking remained significantly higher in LPG-burning huts compared with wood for every subsequent sampling interval, whereas the difference between charcoal and wood declined in later intervals and was no longer significant during the third and fourth intervals.

### Household exit.

Mean household exit was low (8.9% in LPG, 13.3% in charcoal and 10.3% in wood burning huts) and was not significantly different across all three fuel types (Table 2). After adjusting for multiple comparisons, none of the fuels were significantly different from baseline conditions (Figure 3C). Sampling interval was not a significant effect modifier, although exiting was higher in LPG than in wood during interval 2, and higher in charcoal than in wood huts during intervals 2 and 3 (Table 3, Figure 4C).

### Mortality.

Mortality was also low across each fuel type, ranging from 3.8% in LPG huts, to 8.1% in charcoal and 8.7% in wood. This translated to a 40% lower odds of mortality in LPG huts compared with wood (odds ratio = 0.6, 95% CI: 0.4–0.8), whereas charcoal and wood were not significantly different (Table 2). Compared with baseline conditions where no fuel was burned, mortality was higher in wood-burning huts (Figure 3D).

### Effects of PM_2.5_, temperature, and RH.

Average PM_2.5_ concentrations ranged from 29 µg/m^3^ in LPG, 223 µg/m^3^ in charcoal, and 1,672 µg/m^3^ in wood huts (Table 4). PM_2.5_ concentrations in charcoal and wood huts were higher than baseline conditions in which no fuels were burned, whereas LPG was not significantly different. Indoor temperatures were elevated in huts cooking with all three fuels relative to baseline conditions, with the highest temperatures recorded in wood huts (mean = 27°C). Average relative humidity ranged from 56% in charcoal and wood huts to 61% in LPG huts, all of which were significantly higher than the baseline mean of 46% RH.

**Table 4:** Twelve-hour averages of PM_2.5_, temperature, and relative humidity by fuel type

	Fuel	Mean (SD)	Median (IQR)
PM_2.5_ (µg/m^3^)	LPG	29.2 (16.2)	25.1 (16.7)
	Charcoal	149.4 (350.3)***	73.7 (41)
	Wood	1,672.3 (511.5)***	1,759.7 (631.5)
	Baseline (Intercept)	16.1 (5.8)	14.4 (9.3)
Temperature (°C)	LPG	25.7 (1.1)*	25.8 (1.6)
	Charcoal	26.6 (1)*	26.8 (1.1)
	Wood	27.2 (0.9)***	27.2 (1.4)
	Baseline (Intercept)	24.8 (0.8)	25 (0.5)
Relative humidity (%)	LPG	60.6 (5.5)***	60.6 (9.3)
	Charcoal	56.6 (4.2)***	56.2 (4)
	Wood	56.3 (4.5)***	56.1 (6.2)
	Baseline (Intercept)	46.2 (3.7)	45.6 (3.6)

PM_2.5_ = real-time fine particulate matter. Mean and standard deviation (SD) of 12-hour averages for each fuel presented with median and interquartile ranges (IQR). Differences in means for each fuel were compared with baseline values using linear mixed effect regression. *P < 0.05; ***P < 0.001.

Indoor temperature was the most important predictor of household entry, explaining 9% of variance in linear mixed effect models which included PM_2.5_ and RH (Table 5). Each standard deviation increase in temperature was associated with a 13.1 point (95% CI: 10.0–16.2) decrease in the percentage of mosquitoes that entered huts. In contrast, host-seeking appeared to be most influenced by PM_2.5_; PM_2.5_ accounted for 4% of model variance, and each standard deviation increase was associated with a 5.4% point (95% CI: 3.4–7.4) decline in host-seeking. Temperature was the best predictor of household exit, accounting for 16% of model variance when added last to the full model. Each standard deviation increase in temperature was associated with a 3.3 point (95% CI: 2.3–4.4) decline in the percentage of mosquitoes that exited huts. Conversely, higher PM_2.5_ levels were associated with marginal increases in household exit rates. Finally, PM_2.5_ was positively associated with mortality and explained 4% of model variance.

**Table 5:** Effects of PM_2.5_, temperature, and relative humidity on household entry, host-seeking, household exit, and mortality

	Parameter	β (95% CI)	Δ in AIC	Δ in *R*^2^
Household entry	PM_2.5_ (µg/m^3^)	1.2 (–1.8 to 4.1)	−1.2	0.00
	Temperature	−13.1 (–16.2 to −10.0)***	−62.7	0.09
	Relative humidity	−0.7 (–3.9 to 2.5)	−0.9	0.00
	Intercept	44.2 (38.9 to 49.4)		
Host-seeking	PM_2.5_	−5.4 (–7.4 to −3.4)***	−27.5	0.04
	Temperature	0.9 (–1.3 to 3.1)	−0.7	0.01
	Relative humidity	−0.4 (–2.9 to 2.2)	−0.3	0.00
	Intercept	21.6 (16.0 to 27.2)		
Household exit	PM_2.5_	1.1 (0.2 to 2.0)*	−4.3	0.03
	Temperature	−3.3 (–4.2 to −2.3)***	−40.6	0.16
	Relative humidity	0.3 (–0.6 to 1.0)	1.5	0.01
	Intercept	4.5 (2.4 to 6.7)		
Mortality	PM_2.5_	1.3 (0.0 to 2.6)*	−1.0	0.04
	Temperature	0.8 (–0.5 to 2.2)	1.5	−0.01
	Relative humidity	−0.3 (–1.5 to 0.8)	2.9	0.00
	Intercept	6.6 (2.6 to 10.5)		

PM_2.5_ = real-time fine particulate matter. β values represent the linear change in each outcome for a one standard deviation increase in each predictor variable, controlling for all other independent variables. Variables are scaled for comparison. Δ in Akaike information criteria (AIC) shows relative change in model fit when each variable is added last to a full model. Negative values indicate improved model fit. Δ in *R*^2^ is the proportion of variance explained by each variable, calculated as the change in the conditional *R*^2^ value when each variable is added last to the full model. *P < 0.05; **P < 0.01; ***P < 0.001.

**Figure 5.**
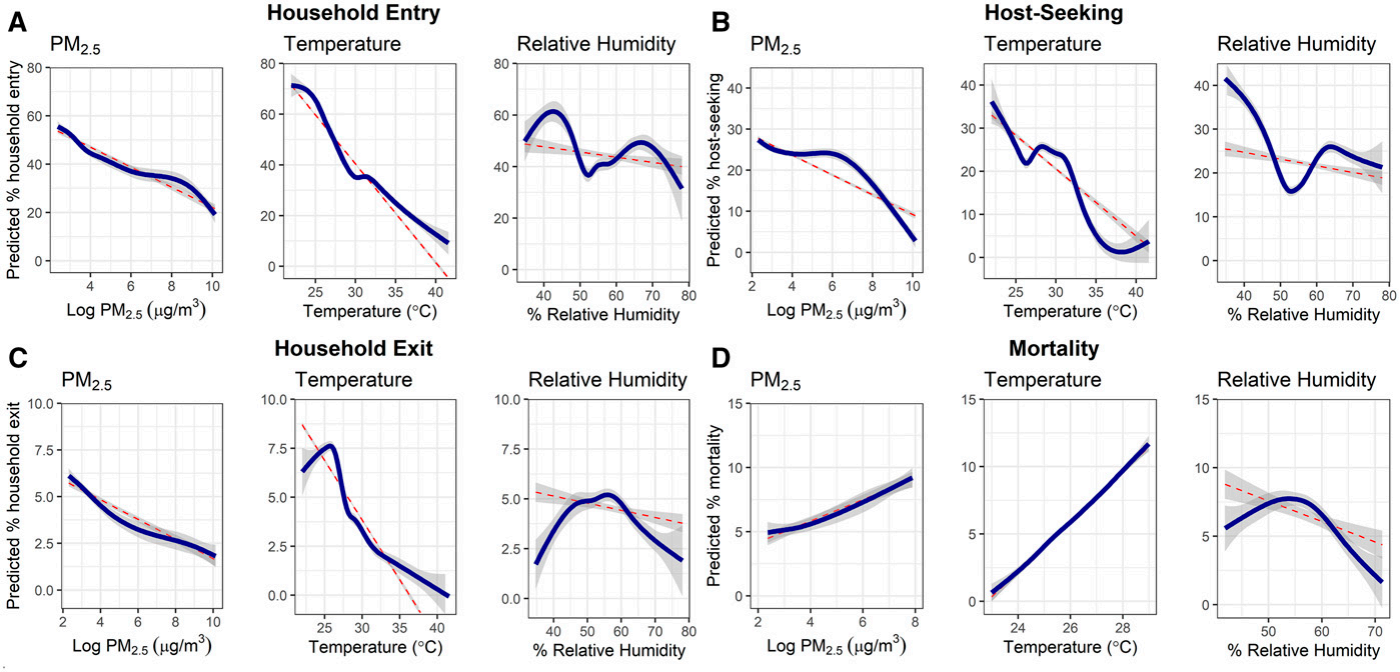
Dose–response effects of PM_2.5_, temperature, and relative humidity on household-entry, host-seeking, household exit, and mortality. Red dotted lines represent predicted values of linear mixed effect models, and solid blue lines represent generalized additive mixed effect model predictions. Gray bands indicated 95% confidence intervals of each model. This figure appears in color at www.ajtmh.org.

Generally, PM_2.5_ and temperature showed linear associations with each outcome. However, the effects of RH on each outcome appeared nonlinear when fitted with GAMMs (Figure 5). Household entry and host-seeking appeared to decline between 50% and 60% RH but then increased above 60% RH. In contrast, household exit and mortality peaked between 50% and 60% RH and declined at lower and higher RH values. After accounting for these nonlinear associations via GAMMs, RH was a significant predictor of host-seeking and household exit, accounting for 5% and 2% of model variance, respectively. Temperature remained the most important predictor of household entry and household exit, and PM_2.5_ was a significant predictor of household entry, host-seeking, and mortality (data not shown).

## DISCUSSION

Under these experimental conditions, the combustion of LPG resulted in increased household entry and host-seeking and reduced mosquito mortality, compared with wood. Charcoal showed a similar pattern when compared with wood, although the differences were less dramatic. Other experimental and observational studies have reported similar effects of biomass fuel combustion on *Anopheles* mosquito behavior and mortality compared with conditions in which no fuels were burned. ^8^^,^ ^10^^–^ ^14^^,^ ^53^ However, this was the first study to explicitly investigate and compare the effects of three commonly used cooking fuels on *Anopheles* mosquito behavior. This information is needed for accurately characterizing potential effects of clean fuel adoption, particularly as LPG is projected to become a dominant fuel in Rwanda and many malaria-endemic countries. ^44^

Given the controlled nature of the experiments, the results are not directly generalizable to field conditions. However, they indicate a potential for clean fuel adoption to result in higher exposure to *Anopheles* mosquitoes via increased household entry and host-seeking compared with houses that cook with biomass fuels. Higher vector density and biting rates are important determinants of malaria and other mosquito-borne disease transmission risk. ^54^^–^ ^57^ Reduced mosquito mortality could also facilitate parasite development and malaria transmission. ^58^ Previous investigations have suggested that reductions in indoor *Anopheles* density in wood-burning houses may be due to higher exiting rates after entry rather than a direct repellent effect of wood fuel combustion. ^17^ However, we observed no differences in household exiting rates across fuel types.

To our knowledge, no other studies have investigated the dose–response effects of PM_2.5_ or other components of fuel combustion on *Anopheles* mosquito behavior. After adjusting for the effects of temperature and RH, PM_2.5_ was a significant predictor of host-seeking and mortality. As has been observed elsewhere, ^59^ temperature was an important determinant of mosquito behavior, particularly household-entry and exit. Although RH was generally less important than temperature and PM_2.5_, it showed significant nonlinear associations with household entry and host-seeking, which both increased above 60% RH. Other studies have also reported increased longevity and fitness of *An. gambiae* at RH levels above 60%. ^60^^,^ ^61^

We observed higher household entry and host-seeking among houses that cooked with LPG compared with baseline conditions where no fuel was used, suggesting a potential attractant effect of LPG fuel. However, PM_2.5_, temperature, and RH explained a relatively low proportion of overall variance in entry and host-seeking. This indicates a potentially important role of other unmeasured components of fuel combustion, such as CO_2_. CO_2_ is primary component of fuel combustion and is the most important attractant for host-seeking mosquitoes. ^5^^,^ ^62^ Increases in ambient CO_2_ levels as little as 0.01% above baseline levels can stimulate female mosquitoes to search for blood meals. ^63^^,^ ^64^ LPG produces more CO_2_ per kilogram of fuel burned than wood or charcoal due to improved combustion efficiency. ^65^ It is conceivable that CO_2_ emissions from LPG attract mosquitoes, whereas components of biomass fuel combustion such as high heat and particulate matter counteract similar effects in wood and charcoal. Average relative humidity was also 14% points higher in LPG huts than in baseline conditions, which could have further attracted host-seeking mosquitoes. Additional experiments are needed to investigate these potential attractant effects of LPG combustion.

A number of factors limit the generalizability of these findings to field settings. In Rwanda, for example, cooking outdoors or in separate kitchen structures is common ^41^ and could have very different effects on mosquito behavior. Other fuelwood species and biomass fuels are also used for cooking, ^37^ each of which could have different effects on mosquitoes. ^8^^,^ ^12^ The smoke exposures measured in this experiment could also exaggerate true exposures under field conditions; individuals may not spend as much time in smoky kitchens as they did in this experiment, and bedrooms are often much further away from kitchens than they were in the experimental huts. Finally, other factors such as proximity to breeding sites, housing characteristics, or use of mosquito control interventions could be more important than cooking fuels in influencing vector density and human exposure. ^31^^,^ ^66^^–^ ^68^ Further experimental studies could explore the impacts of cooking characteristics (e.g., cooking location and fuelwood types) on the behavior of mosquitoes. Well-designed field studies are also needed to measure possible impacts under real-world conditions where other determinants of vector bionomics could vary widely.

We measured PM_2.5_ as a proxy for smoke. However, our ability to draw conclusions about direct effects of PM_2.5_ is limited because we were unable to measure other elements of fuel combustion such as CO_2_, CO, or chemical volatiles. Additionally, we were not able to calibrate the nephalometric PATS+ devices against gravimetric readings, and some readings were outside the limits of detection set by the manufacturer. PM_2.5_ measurements should be therefore being interpreted as relative concentrations between each fuel type rather than exact values.

We used laboratory-reared *An. gambiae* s.s., Kisumu strain mosquitoes to eliminate potential health risks and confounding associated with conducting the experiment with wild mosquitoes. However, laboratory-reared insects may be less robust than their wild counterparts, ^69^ and it is unknown whether wild *Anopheles* mosquitoes would display the same behaviors as those used in this study. We are also unable to generalize to other *Anopheles* species, nor to other important vector genera such as *Culex* and *Aedes* mosquitoes. However, other studies have reported repellant and deterrent effects of biomass combustion on species within these genera. ^11^^,^ ^13^^,^ ^70^^,^ ^71^

Changes in cooking fuels could also indirectly influence malaria risk independently of their direct effects on vector behavior. For example, less smoky fuels could reduce the need for frequent net washing, which could reduce net deterioration and prolong insecticidal activity. ^72^^,^ ^73^ LPG adoption can lead to changes in cooking behavior or time spent indoors, ^39^^,^ ^74^ which could affect exposure to insect vectors independently of the actual type of fuel used. Reductions in HAP exposure could also improve innate immune function ^75^^,^ ^76^ and reduce susceptibility to malaria or other infectious diseases. Conversely, a recent cohort study from Ghana showed that malaria can attenuate the health benefits of reduced HAP exposure; reduced CO resulted in improved growth for infants born to mothers with no evidence of placental malaria, whereas the same effect was not observed if mothers had placental malaria. ^77^ Epidemiological studies should accompany entomological efforts to better characterize the overall effects of clean fuel adoption on risk of malaria and other vectorborne diseases.

## CONCLUSION

Our study suggests that cooking fuels can have important impacts on mosquito behavior. Huts cooking with LPG saw higher household entry and host-seeking, coupled with reduced mosquito mortality. This implies that, at least in highly controlled conditions, the adoption of cleaner fuels could reduce or reverse repellant and deterrent effects from biomass fuels, potentially altering human exposure to *Anopheles* mosquitoes and the pathogens they can transmit. If these findings are confirmed in larger studies under field conditions, these implications would make it incumbent on program implementers to address the increased exposure to disease vectors that may be associated with adoption of cleaner cooking fuels.

Despite these findings, the benefits of clean cooking fuels almost certainly outweigh potential risks from potentially associated changes in vector behavior. Household air pollution is responsible for 1.8 million deaths each year, and the promotion of HAP reduction interventions should remain a public health priority. ^2^^,^ ^78^ At the same time, further entomological and epidemiological studies should be conducted to better characterize changes associated with clean fuel adoption and their potential to affect incidence of malaria or other vectorborne diseases. If the risk is indeed elevated, enhanced vector-control interventions could be promoted in tandem with cleaner cooking fuels. For example, a growing line of research is investigating built-environment solutions such as house screening for reducing exposure to disease vectors. ^79^ These strategies could be paired with clean cooking interventions as part of an overall approach to improving household environmental health. ^80^
